# Hemorrhage Exacerbates Radiation Effects on Survival, Leukocytopenia, Thrombopenia, Erythropenia, Bone Marrow Cell Depletion and Hematopoiesis, and Inflammation-Associated microRNAs Expression in Kidney

**DOI:** 10.1371/journal.pone.0139271

**Published:** 2015-09-30

**Authors:** Juliann G. Kiang, Joan T. Smith, Marsha N. Anderson, Joshua M. Swift, Christine L. Christensen, Paridhi Gupta, Nagaraja Balakathiresan, Radha K. Maheshwari

**Affiliations:** 1 Radiation Combined Injury Program, Armed Forces Radiobiology Research Institute, Uniformed Services University of the Health Sciences, Bethesda, Maryland, United States of America; 2 Department of Radiation Biology, Uniformed Services University of the Health Sciences, Bethesda, Maryland, United States of America; 3 Department of Medicine, Uniformed Services University of the Health Sciences, Bethesda, Maryland, United States of America; 4 Department of Military and Emergency Medicine, Uniformed Services University of the Health Sciences, Bethesda, Maryland, United States of America; 5 Undersea Medicine Department, Naval Medical Research Center, Silver Spring, Maryland, United States of America; 6 Comparative Pathology Division, Veterinary Sciences Department, Armed Forces Radiobiology Research Institute, Uniformed Services University of the Health Sciences, Bethesda, Maryland, United States of America; 7 Department of Pathology, Uniformed Services University of the Health Sciences, Bethesda, Maryland, United States of America; National Cancer Institute, UNITED STATES

## Abstract

Exposure to high-dose radiation results in detrimental effects on survival. The effects of combined trauma, such as radiation in combination with hemorrhage, the typical injury of victims exposed to a radiation blast, on survival and hematopoietic effects have yet to be understood. The purpose of this study was to evaluate the effects of radiation injury (RI) combined with hemorrhage (*i*.*e*., combined injury, CI) on survival and hematopoietic effects, and to investigate whether hemorrhage (Hemo) enhanced RI-induced mortality and hematopoietic syndrome. Male CD2F1 mice (10 weeks old) were given one single exposure of γ- radiation (^60^Co) at various doses (0.6 Gy/min). Within 2 hr after RI, animals under anesthesia were bled 0% (Sham) or 20% (Hemo) of total blood volume via the submandibular vein. In these mice, Hemo reduced the LD50/30 for 30-day survival from 9.1 Gy (RI) to 8.75 Gy (CI) with a DMF of 1.046. RI resulted in leukocytopenia, thrombopenia, erythropenia, and bone marrow cell depletion, but decreased the caspase-3 activation response. RI increased IL-1β, IL-6, IL-17A, and TNF-α concentrations in serum, bone marrow, ileum, spleen, and kidney. Some of these adverse alterations were magnified by CI. Erythropoietin production was increased in kidney and blood more after CI than RI. Furthermore, CI altered the global miRNAs expression in kidney and the ingenuity pathway analysis showed that miRNAs viz., let-7e, miR-30e and miR-29b that were associated with hematopoiesis and inflammation. This study provides preliminary evidence that non-lethal Hemo exacerbates RI-induced mortality and cell losses associated with high-dose γ-radiation. We identified some of the initial changes occurring due to CI which may have facilitated in worsening the injury and hampering the recovery of animals ultimately resulting in higher mortality.

## Introduction

Radiation exposure events in our history have demonstrated that irradiated victims are also often subjected to other trauma such as hemorrhage, wounds, burns, brain injuries, or bone fractures. A significant number of these combined traumas were observed after the bombings at Hiroshima and Nagasaki [1, 2]. In addition, some of victims at the Chernobyl reactor meltdown were exposed to radiation plus another trauma [3]. In rodent models of combined trauma, burns and wounds increase morbidity and mortality after otherwise nonlethal radiation exposures [4–6]. Secondary consequences to radiation combined with wound or burn in survivors of these injuries plus others are known to show exacerbated acute radiation syndrome, including enteropathy associated with hematopoietic syndrome [7, 8]. Our laboratory reports that the combined trauma of non-lethal hemorrhage following non-lethal ionizing radiation is detrimental to bone and bone marrow more than either injury alone [9]. This loss of bone mass acutely following radiation exposure is due to immediate elevations in osteoclast activation and activity [10] coupled with decreased osteoblast activity [11], resulting in reduced bone formation activity and volume [12]. These negative changes on bone present long-term health complications in first responders and survivors of a nuclear attack, nuclear accident, or exposure to a radioactive dispersal device (RDD) [13,14].

Either lethal hemorrhage (Hemo) or lethal radiation injury (RI) results in similar outcomes. Hemo at 40% of total blood volume increases IL-10 and TNF-α concentration in blood, NF-κB activation and iNOS expression in murine small intestine, and cell apoptosis in various murine tissues [15–18]. Likewise, sub-lethal ionizing radiation also increases these parameters [4] in addition to initiating deleterious hematopoietic changes [8]. It was not clear whether non-lethal Hemo affected ionizing RI-induced outcomes. MicroRNAs (miRNAs) are small, endogenous noncoding RNAs that post transcriptionally regulate gene expression. MiRNAs are involved in various biological processes such as hypoxia, differentiation, inflammation, cell proliferation, cell death, and fibrosis in kidney disease [19]. However, miRNA expression pattern in CI and their role in hematopoesis and inflammation are unknown. In this study we tried to understand CI specific pathophysiology due to miRNA modulation. Understanding whether the synergistic effects of these two traumas on survival and hematopoiesis is present is imperative to determine preventative measures that can be utilized to save lives under scenarios of nuclear accidents.

The aim of this study was to determine whether non-lethal Hemo, when combined with sub-lethal ionizing radiation (i.e. combined injury, CI), exacerbated the effects of radiation exposure on hematopoiesis and mortality. The former was regulated by NF-κB and miRNAs. We hypothesized that CI would be more detrimental to bone marrow than either assault alone. Mice were used to test the hypothesis and achieved the objective, because studies with a whole animal covered interactions among organs and molecular components of transcription factors, cytokines and microRNA.

## Materials and Methods

### Ethics statement

Research was conducted in a facility accredited by the Association for Assessment and Accreditation of Laboratory Animal Care-International (AAALACI). All procedures involving animals were reviewed and approved by the AFRRI Institutional Animal Care and Use Committee. Euthanasia was carried out in accordance with the recommendations and guidelines of the American Veterinary Medical Association. For the survival study, we observed animals every 2 hours during work hours, and moribund animals were euthanized according to humane endpoints. The clinical definition of moribund is being in the state of dying with no expectation of recovery, where animals display a combination of the following: lowered body temperature, slow or impaired motion, continuous shaking, hunched back, and inability to maintain sternal recumbency. Moribund animals were placed in a separate cage where carbon dioxide gas was applied until no breathing was observed, followed by a cervical dislocation as a secondary confirmatory method of euthanasia. Deceased animals were immediately removed from the cages to avoid any health problems caused by means other than the experimental treatment. Any surviving animals at the end of the study were subjected to euthanasia, also by the application of carbon dioxide followed by cervical dislocation. For the studies other than those testing survival, mice at specific endpoints were placed under anesthesia by isoflurane inhalation for the entire period of blood collection, immediately followed by a confirmatory cervical dislocation for euthanasia and terminal tissue collection.

### Animals and experimental design

Male CD2F1 mice (10 weeks old), were obtained from Harlan Labs (Indianapolis, IN) and allowed to acclimate to their surroundings for 14 days prior to initiation of the study. All animals were randomly group housed in a temperature (68–75°F)- and light-controlled room (12-hr light-dark cycle) and randomly divided to 4 experimental groups (N = 16/group for survival experiments and N = 6/group for mechanistic elucidation): Sham (0 Gy), Hemorrhage (Hemo, 20% total blood volume), Radiation Injury (RI), or RI+Hemo. After injuries, mice were assigned to clean cages with 2–4 mice/cage and provided with proper food (standard rodent chow, Harlan Teklad 8604) and acidified water *ad libitum*. The health status of animals was monitored daily and the research was conducted in a facility accredited by the Association for Assessment and Accreditation of Laboratory Animal Care-International (AAALACI). All procedures involving animals were reviewed and approved by the Armed Forces Radiobiology Research Institute (AFRRI) Institutional Animal Care and Use Committee (IACUC).

### Radiation injury (RI)

Mice were placed in well-ventilated acrylic restrainers and one whole-body dose of ^60^Co γ-photon radiation was delivered at a dose rate of approximately 0.6 Gy/min. Dosimetry was performed using the alanine/electron paramagnetic resonance system. Calibration of the dose rate with alanine was traceable to the National Institute of Standards and Technology and the National Physics Laboratory of the United Kingdom. Sham-irradiated mice were placed in the same acrylic restrainers, taken to the radiation facility, and restrained for the time required for irradiation.

### Hemorrhage (Hemo)

Within 2 hours post-RI, mice were anesthetized under isoflurane (∼3%) and bled 0% (Sham, RI) or 20% (Hemo, RI+Hemo) of total blood volume via the submandibular vein as previously described [20]. Briefly, the jaw of the anesthetized mouse was cleaned with a 70% EtOH wipe, and glycerol was applied to the surface of the jaw to allow for ease of collection and measurement of blood loss. A 5 mm Goldenrod animal lancet (MEDIpoint, Inc; Mineola, NY) for facial vein blood samples was used to puncture the submandibular vein of the mouse and heparinized hematocrit collection tubes (75 mm; Drummond Scientific Co.; Broomall, PA) were marked and used to collect the appropriate amount of blood to ensure 20% of total blood volume was extracted during the hemorrhage process. The volume of blood collected was based upon each individual mouse’s body mass [21].

### Survival

Survival studies (N = 16/group) were performed with 16 mice per group. Gross appearance, general health, and survival of each mouse were followed by visual inspection 3–4 times daily for 30 days, as previously described [8].

### Water consumption

Drinking water was provided in a steam-sterilized graduated bottle and placed on the top of each cage, in which 4 mice were housed. The drinking bottle was connected to a sipping tube with a metal ball inside to prevent water leakage. The volume of water was measured daily for the first 7 days. The average volume drank per mouse per day was calculated [8]. Data were expressed as mL/animal/day.

### Body weight

The body weight of each mouse was measured at the start of the experiments and on days 1, 3, 7, 14, 15, 21, 28 and 30 after hemorrhage, radiation, and radiation followed by hemorrhage. Data were expressed as g.

### Assessment of blood cell profile in peripheral blood

Whole blood was collected by terminal cardiac puncture from mice (N = 6/group per time point) anesthetized by isoflurane (∼3%) on various time points after RI or CI and placed in EDTA tubes and assessed with the ADVIA 2120 Hematology System (Siemens, Deerfield, IL). Differential analysis was conducted using the peroxidase method and the light scattering techniques recommended by the manufacturer [8]. Data were expressed as cells/mL.

### Measurements of bone marrow cells

Bone marrow cells from femurs (N = 6 per group) were collected at various time points after RI or CI by flashing marrows out with 3 ml 1x phosphate-buffered saline (PBS) buffer twice. The cells were then centrifuged at 800 × g for 6 min, re-suspended in 10 ml 1x PBS buffer, and then placed in CountessTM cell-counting-chamber slides (Invitrogen) and counted using a Countess automated cell counter (Invitrogen) [8]. Data were expressed as cells/femur. Bone marrow cells were centrifuged again at 800 x g for 6 min. The cell pellets were stored in -70°C until use.

### Histopathology assessment

Sternum specimens were collected for histopathology 3 and 15 days after RI or CI (N = 3 per group). Specimens were immediately fixed in 10% phosphate-buffered formalin upon removal. The tissue was then embedded in paraffin, sectioned transversely and stained with hematoxylin and eosin (H&E). Microscopic evaluation was performed by a board certified veterinary pathologist. Tissue imaging was performed using a NanoZoomer 2.0 from HAMAMATSU PHOTONICS K.K. (Hamamatsu, Japan). The same acquisition setting, including scaling, applies to all images in the same figure.

### Tissue lysates

Bone marrow cells collected from 2 femurs, ileum, spleen, and kidney tissues (N = 6/group) were mixed with Na^+^ Hanks’ solution, sonicated for 15 s, and then centrifuged at 10,000 x g for 10 min. Supernatants were conserved for protein determination and stored at -70°C until use.

### Western blots

Whole caspase-3 protein levels in bone marrow cells, HIF-1α, NF-κB-p50, NF-κB-p52, NF-κB-p65, RelB, and IgG in kidney lysates were determined. Total protein in the cell lysates was determined with Bio-Rad reagent (Bio-Rad; Richmond, CA). Samples with 20 μg of protein in tris buffer (pH = 6.8) containing 1% sodium dodecyl sulfate (SDS) and 1% 2-mercaptoethanol were resolved on SDS-polyacrylamide slab gels (Novex precast 4–20% gel; Invitrogen; Carlsbad, CA). After electrophoresis, proteins were blotted onto a PVDF membrane (0.45 μm; Invitrogen), using a Novex blotting apparatus and the manufacturer's protocol (Invitrogen). After blocking the nitrocellulose membrane by incubation in tris-buffered saline-0.5% tween20 (TBST) containing 3% nonfat dried milk for 90 min at room temperature, the blot was incubated for 60 min at room temperature with antibodies directed against caspase-3 (AbCam), HIF-1α, NF-κB-p50, NF- κ B-p52, NF-κB p65, RelB, and IgG (Santa Cruz Biotechnology; Santa Cruz, CA) at a concentration of 1 μg/ml in TBST—3% dry milk. The blot was then washed 3 times (10 min each) with TBST before incubating the blot for 60 min at room temperature with a 1000x dilution of species-specific IgG peroxidase conjugate (Santa Cruz Biotechnology) in TBST. The blot was washed 6 times (5 min each) in TBST before detection of peroxidase activity using the Enhanced Chemiluminenscence Plus (Amersham Life Science Inc., Arlington Heights, IL, USA). IgG levels were not altered by radiation and were used as a control for protein loading. Protein bands of interest were quantitated densitometrically and normalized to IgG. Data were expressed as intensity ratio to IgG, because IgG levels present in tissues was not affected after irradiation [4].

### Activated caspase-3 measurements

Activated caspase-3 protein levels were measured using Quantikine ELISA kit according to the manufacturer’s protocol (R&D SYSTEM, Minneapolis, MN).

### RNA isolation and quantitation

Total RNA including miRNA was isolated from kidney samples using the mirVana miRNA isolation kit (Cat#AM1560; Life Technologies, Carlsbad, CA, USA), according to the manufacturer's protocol. Briefly, 20x volume of lysis buffer was added to the sample and homogenized on ice followed by adding 1/10th volume of homogenate additive to the lysate. Samples were incubated at 4°C for 10 min and equal volume of phenol:chloroform was added to the tissue lysate, vortexed, and centrifuged for 8 min at 12,000 × g. The aqueous layer was collected after centrifugation and mixed with 1.25 volume of absolute ethanol and passed through the RNAqueous micro kit cartridge. The flow through was discarded and the column was washed once with 700 μL of wash solution-1, twice with 500 μL of wash solution-2/3. RNA was finally eluted in pre-heated RNAse-free water.

Both quality and quantity of the miRNA in the total RNA isolated were measured and analyzed using Agilent Small RNA kit (Cat#5067–1548; Agilent Technologies, Santa Clara, CA, USA) in Agilent 2100 Bioanalyzer.

### MiRNA profiling

Kidney total RNA containing 5 ng of miRNA was reverse transcriped (RT) with TaqMan miRNA RT Kit (Life Technologies, Carlsbad, CA, USA) as described before [22]. Briefly, RT was performed using RNA sample with megaplex pools of stem-loop RT primers for pool A and B; and TaqMan microRNA reverse transcription kit (Applied biosystems Inc., Carlsbad, CA). RT reaction mixture contained 0.8 μl Megaplex RT primers Rodent Pool A/B (v2.0), 0.2 μl 100 mM dNTPs (with dTTP), 1.5 μl Multiscribe reverse transcriptase (50 U/μl), 0.8 μl 10× RT Buffer, 0.9 μl MgCl_2_ (25 mM), 0.1 μl RNAse inhibitor (20 U/μl), RNA template and nuclease free water to a final volume of 7.5 μl. RT reaction was carried out on Veriti 96-Well Thermal Cycler (Life Technologies, Carlsbad, CA, USA) with the following reaction conditions: [16°C/2min; 42°C/1min; 50°C/1sec] X 40 cycles; 85°C /5 min; and hold at 4°C. Pre-amplification of RT products was performed using the pre-amplification master mix and primer set (Life Technologies, Carlsbad, CA, USA) as per manufacturer’s protocol with the following reaction conditions: 95°C/10min; 55°C/2min; 72°C/2min; [95°C/15sec; 60°C/4min] X 16 cycles; 99°C/10min; and held at 4°C. The undiluted pre-amplification products were used for the miRNA profiling using TaqMan Low-Density Rodent microRNAs Array (TLDA) Set v2.0 (Applied Biosystems, Inc) containing 561 rodent miRNAs as per manufacturer’s protocol. The quantitative RT-PCR (qRT-PCR) reaction was carried out at default thermal-cycling conditions in ABI 7900HT Fast Real-Time PCR System (Applied Biosystems, Life Technologies, Foster City, CA).

### Data analysis of miRNA array

Real-time PCR raw data were analyzed using the Real-Time StatMiner® Software V.4.5.0.7 (Integromics, Madison, WI). The data were normalized to U6 snRNA as an optimal endogenous control. Relative quantitation (RQ) of miRNA expression between control and injured groups was done by filtering of miRNAs having expression Ct values below 35 cycles and the detection of expression in all biological replicates of calibrator and target. The miRNAs that had more than 2.0 fold modulation with a P value <0.05 were considered as significantly modulated miRNAs. Predicted targets of differentially expressed kidney miRNAs downloaded from miRWalk, a target prediction algorithm, were analyzed. Both functional and network analysis of altered miRNA and their gene targets were performed using Ingenuity Pathway Analysis (IPA) program (Ingenuity Systems Inc, Redwood City, CA).

### Cytokine and chemokine measurements

Mice (N = 6 per group) were anesthetized by isoflurane 1 day after CI. Their whole blood was collected by terminal cardiac puncture. CapiJect tubes (Terumo; Somerset, NJ) were used to separate sera by centrifugation at 3,500 g for 90 sec and stored at -70°C until assayed. Cytokine concentrations in serum, bone marrow, ileum, spleen, and kidney lysates were analyzed using the Bio-PlexTM Cytokine Assay (Bio-Rad; Hercules, CA) following the manufacturer’s directions. Briefly, serum from each animal was diluted fourfold and examined in duplicate. Data were analyzed using the LuminexH 100TM System (Luminex Corp.; Austin, TX) and quantified using MiraiBio MasterPlexH CT and QT Software (Hitachi Software Engineering America Ltd.; San Francisco, CA), and concentrations were expressed in pg/mL unless otherwise noted. The cytokines reported herein were interleukin (IL)-1β, IL-6, IL-17A, KC, Rantes, and TNF-α [8]. Data were expressed as pg/mL in serum and pg/mg protein in tissues.

### Statistical analysis

Sixteen mice per group were used for each survival experiment to gain statistical significance by Mantel-Cox procedure (total N = 16 per group). All other results are expressed as means ± SEM (N = 6 per group). ANOVA, Bonferroni’s inequality, and Student’s *t*- test were used for comparison of groups. For all data, statistical significance was accepted at p<0.05 or otherwise indicated.

## Results

### CI reduces survival and water consumption

**Fig 1:**
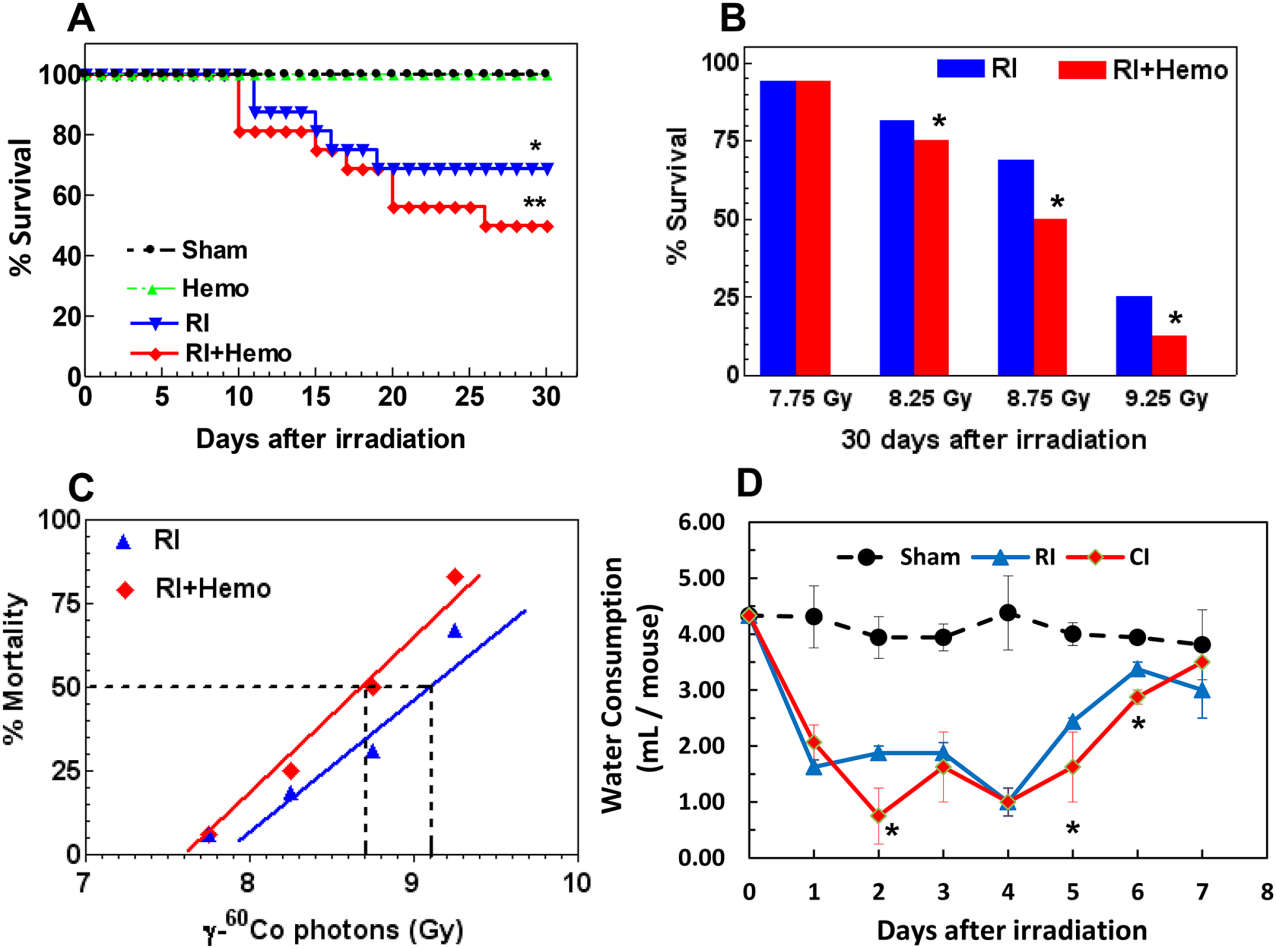
CI reduces survival and water consumption. Mice received 7.75–9.25 Gy radiation and/or 20% hemorrhage to establish following four treatment groups: Sham, Hemo RI, or RI+Hemo. All values are means ± SEM. **(A)** No mice died in Sham and Hemo groups (data not shown). Mice received 8.75 Gy, N = 16 per group. *p<0.05 vs. Sham or Hemo group; **p<0.05 vs. RI, determined by Mantel-Cox test. **(B)** Mice received 7.75 Gy, 8.25 Gy, 8.75 Gy, or 9.25 Gy, N = 16 per group. Their survival was monitored for 30 days. *p<0.05 vs. respective RI group, determined by Mantel-Cox test. **(C)** Mortality in % in function of radiation doses was graphed and the 50% lethal radiation dose over 30 days (LD50/30) was calculated. Their LD50/30s are 8.7 Gy for RI and 9.1 Gy for RI+Hemo, respectively, and DMF is 1.046. **(D)** Mice received 9.25 Gy, N = 16 per group. Their daily water consumption for first 7 days after RI was measured. The average volume of daily water consumption is 4.081 ± 0.084 mL/mouse (N = 16 over a 7-day period of time). *p<0.05 vs. respective RI time point, determined by Student’s *t*-test. RI: radiation injury; Hemo: 20% bleeding; Sham: free of any injury.

RI-induced mortality, in mice, begins to occur 10 days after exposure to ionizing radiation [8]. RI combined with 15% skin-wound trauma (RI-W CI) induces an earlier onset and an enhancement of mortality [8]. Therefore, we tested whether RI combined with non-lethal Hemo (CI) would induce a similar outcome. Indeed, as shown in Fig 1A, CI mice began to die on day 9 after irradiation, whereas death was noted to begin on Day 10 after RI. The increased mortality was observed between day 20 and day30. Hemo alone did not produce any mortality. By Day 30, CI induced more mortality than RI alone (Fig 1B) with the LD_50/30_ 8.7 Gy for CI and 9.1 Gy for RI (Fig 1C). The dose modifying factor (DMF) is 1.046.

We have previously noted that mice exposed to Radiation-Wound combined injury (R-W CI) drink more water, on average, than the sham-exposed mice [8]. Therefore, water consumption was measured daily for the first 7 days after CI. The average volume of daily water consumption is 4.081 ± 0.084 mL/mouse (N = 16 over a 7-day period of time). Although Hemo alone reduced the daily water consumption only on the first day after Hemo (S1 Fig), Fig 1D depicts that CI mice drank water less than RI mice on days 2, 5, and 6 (p<0.05 vs. RI group).

We selected 8.75 Gy (LD_50/30_ for CI) for the subsequent mechanistic elucidation, because the dose was studied with this mouse strain in the literature [23, 24].

### CI enhances body weight loss

**Fig 2:**
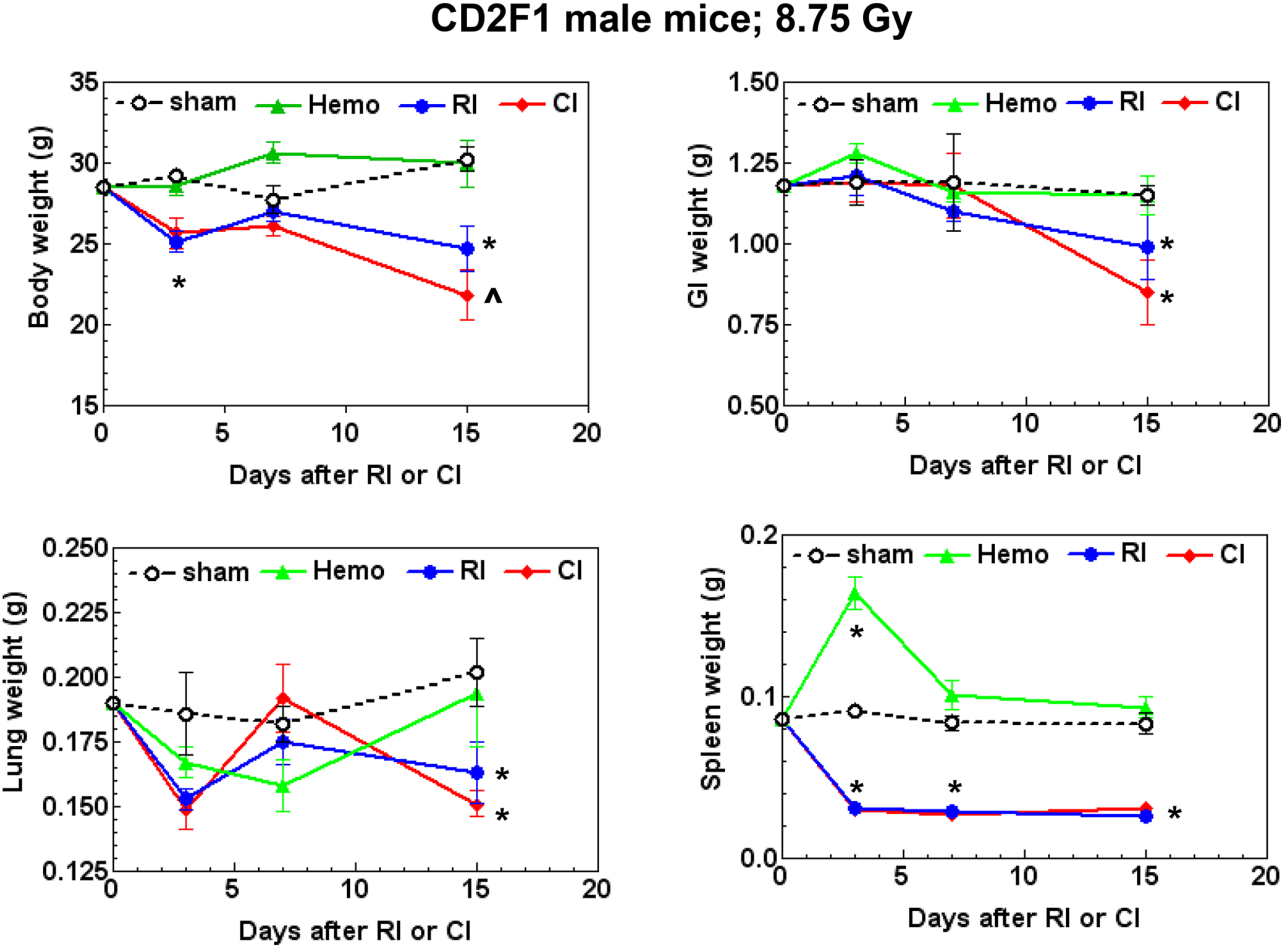
CI enhances body weight loss. Mice received 8.75 Gy. Body weight, small intestine weight, lung weight, and spleen weight were measured on 4–5 hr, 3, 7, and 15 days after RI (N = 6 per group at each time point). All values are means ± SEM. *p<0.05 vs. sham and Hemo; ^p = 0.08 vs. respective RI time point, determined by Student’s *t*-test. Sham: free of any injury; Hemo: 20% bleeding; RI: radiation injury; CI: RI+Hemo.

It is known that RI induced bodyweight loss that is enhanced after R-W CI [8]. We have reported that wound alone did not change the body weight compared to the sham control, while the irradiated and combined injured animal survival curves were parallel with the body weight by day 15. Then the remaining surviving animals begin to gain weights up to day 30, the end of the observation period. Like R-W CI, similar results were observed after sham, Hemo, RI, and CI (S2 Fig). Therefore, the time-course study for cellular and molecular changes was up to day 15. We measured body weight after CI at 8.75 Gy. On day 15, as depicted in Fig 2A, CI resulted in a -12% greater reduction in body mass as compared to RI alone (p<0.05). In addition, both RI and CI induced significant reductions, respectively, in GI (-26%; -14%), lung (-19%; 25%), and spleen (68%; 62%) weights as compared to both Sham and Hemo groups (Fig 2B–2D).

### CI enhances WBC, RBC, and platelet reductions

**Fig 3:**
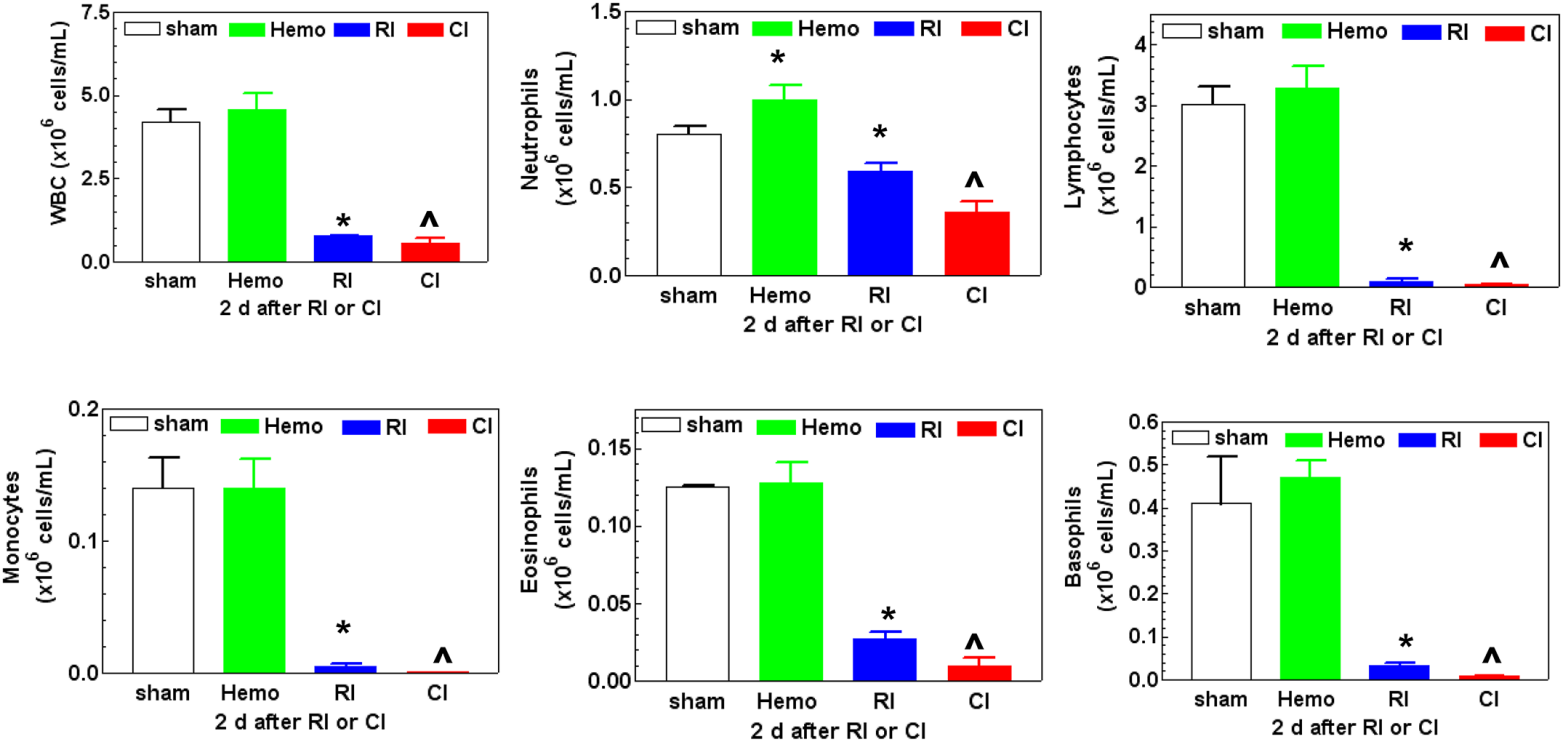
CI enhances WBC loss. Mice received 8.75 Gy. Bloods were collected at various time points after RI (N = 6 per group at each time point). Hematological analysis was presented with samples on day 2 after RI. All values are means ± SEM. *p<0.05 vs. sham and Hemo; ^p<0.05 vs. respective RI time point, determined by Student’s *t*-test. Sham: free of any injury; Hemo: 20% bleeding; RI: radiation injury; CI: RI+Hemo.

It is known that RI induced WBC loss that was enhanced by day 1 after R-W CI [8]. Therefore, we measured white blood cell (WBC) counts after CI in this model. Hemo alone reduced WBC counts on day 1 (-23% vs. Sham, p<0.05), but then fully recovered on day 2. RI reduced WBC counts within 4 hr after RI (-70% vs. Sham, p<0.05), continued to reduce through day 3 (-97% vs. Sham, p<0.05), and then remained at this level through day 15. Similarly to RI, CI also reduced WBC counts within 4 hr after CI, but on days 1 and 2, the CI-induced WBC reduction was greater than RI. Fig 3 shows that RI significantly decreased WBC counts by day 2 (-81% vs. Sham, p<0.05), mainly due to losses of neutrophils, lymphocytes, monocytes, eosinophils, and basophils. CI resulted in further reductions in WBC measurements (-86% vs. Sham, p<0.05). This CI enhancement was not observed on days 3, 7, or 15.

**Fig 4:**
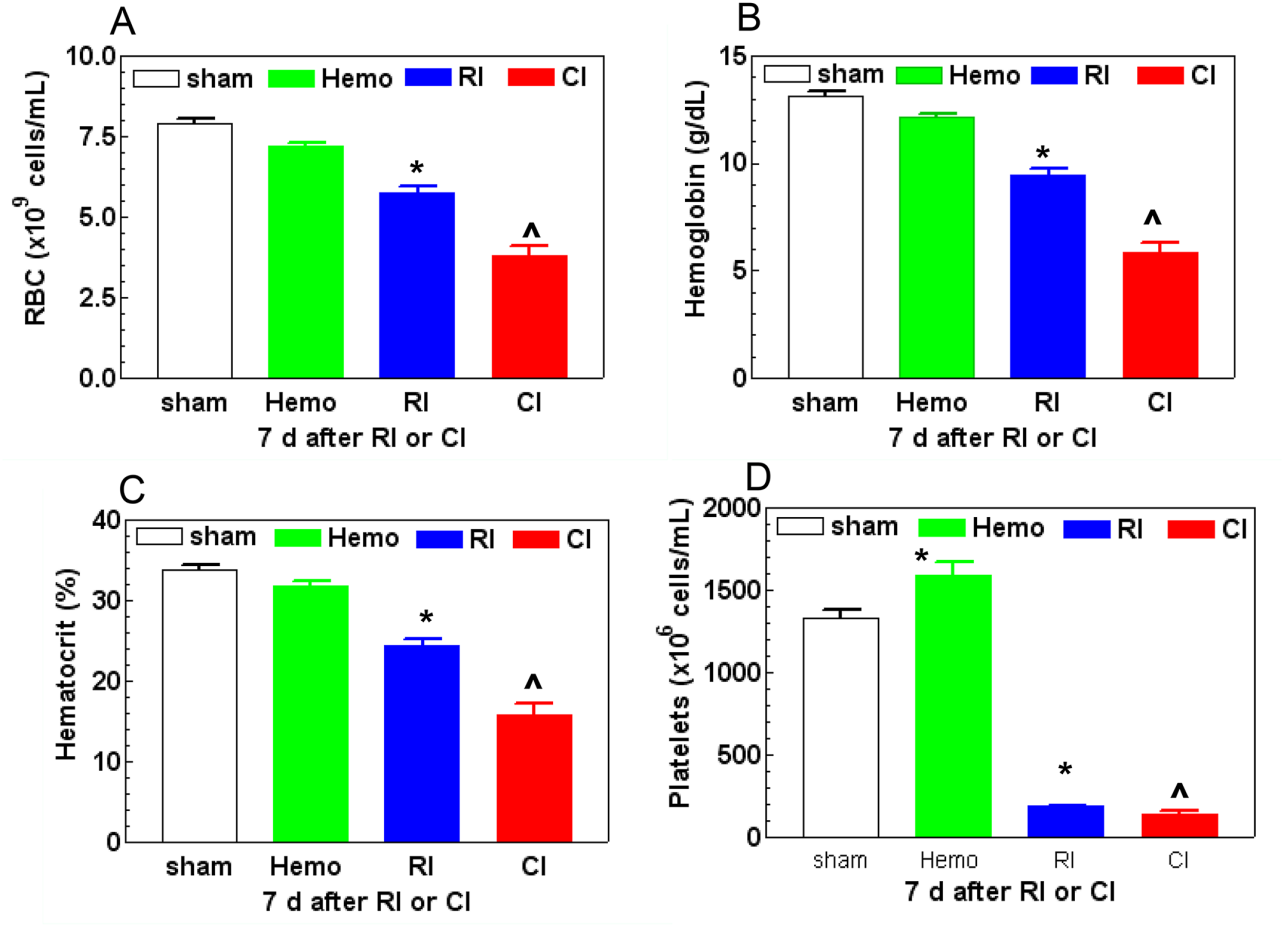
CI enhances RBC and platelet loss. Mice received 8.75 Gy. Bloods were collected at various time points after RI (N = 6 per group at each time point) and hematological analysis was presented with samples on day 7 after RI. All values are means ± SEM. *p<0.05 vs. sham and Hemo; ^p<0.05 vs. respective RI time point, determined by Student’s *t*-test. Sham: free of any injury; Hemo: 20% bleeding; RI: radiation injury; CI: RI+Hemo.

In addition to alterations in WBC counts, Hemo alone reduced RBC counts within 4 hr (-25% vs. Sham, p<0.05), but then fully recovered on day 15. Unlike Hemo, RI decreased red blood cell (RBC) counts on day 7 (-27% vs. Sham, p<0.05) and continued to reduce on day 15 after RI (-54% vs. Sham, p<0.05). CI resulted in further reductions in RBC counts as compared to RI mice on days 7 through 15 (-51% to -76% vs. Sham, p<0.05). Fig 4A–4C shows that CI resulted in a further reduction on day 7; RBC, hemoglobin, and hematocrit readings levels were significantly reduced on day 7 after RI and CI.

Hemo reduced platelet counts within 4 hr (-24% vs. Sham, p<0.05), but fully recovered on day 1. Then, Hemo caused increases in platelet counts on day 2 through day 7 (+17% to +19% vs. Sham, p<0.05), and then returned to the baseline on day 15. RI-induced decreases in platelet counts were observed on day 7 through day 15 (-86% to -98% vs. Sham, p<0.05, Fig 4D). CI resulted in further reductions in platelet counts on day 7 (-90% vs. Sham, p<0.05, Fig 4D). But this CI-induced enhancement of platelet reduction disappeared by day 15.

### CI enhances bone marrow cell loss but not activated caspase-3 protein

**Fig 5:**
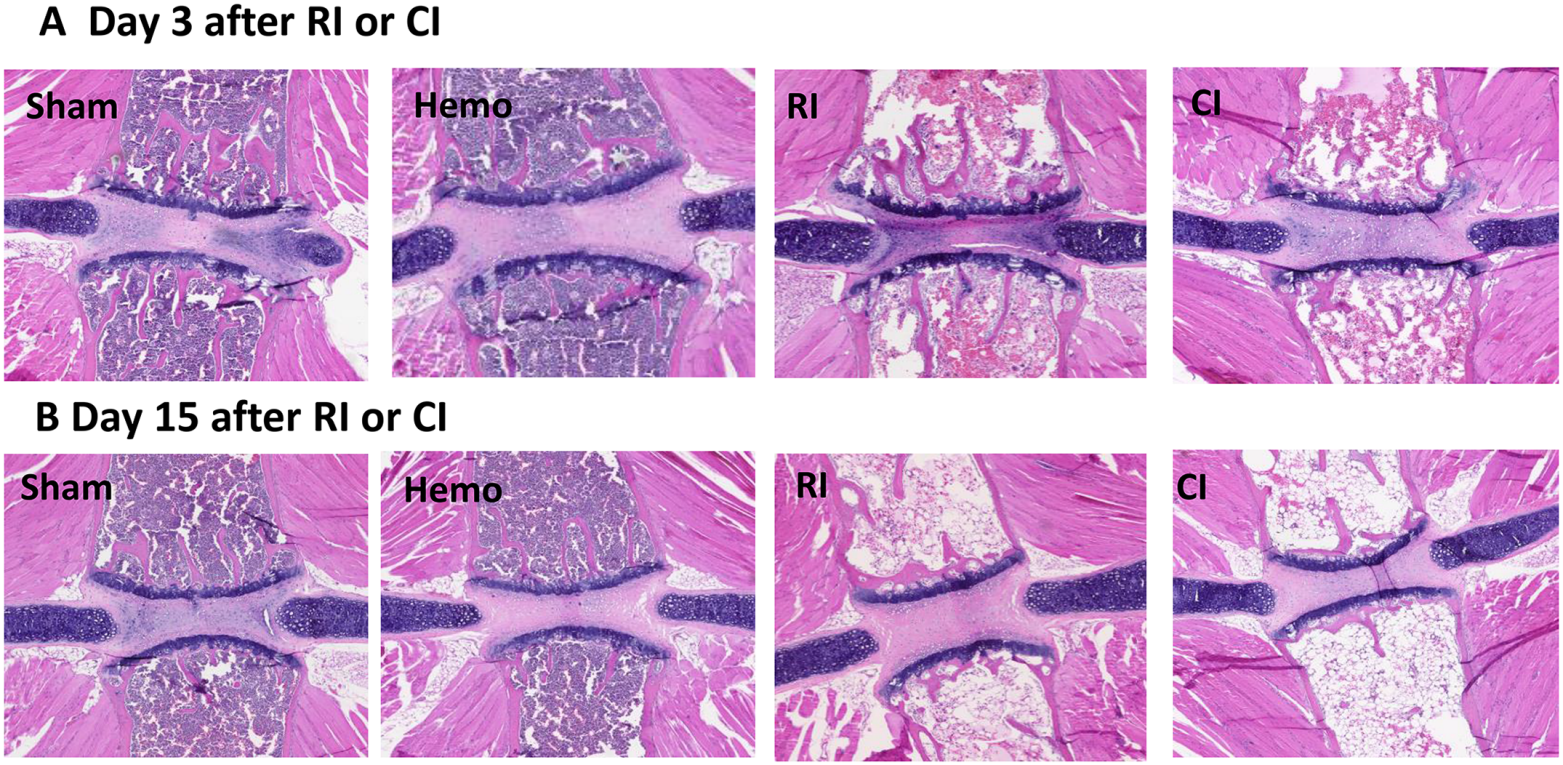
RI and CI reduce bone marrow cellularity. Mice received 8.75 Gy. Sternums were collected at various time points after RI or CI for histology stained with H&E. n = 3 per group at days 3 and 15 after RI or CI. Sham: free of any injury; Hemo: 20% bleeding; RI: radiation injury; CI: RI+Hemo.

**Fig 6:**
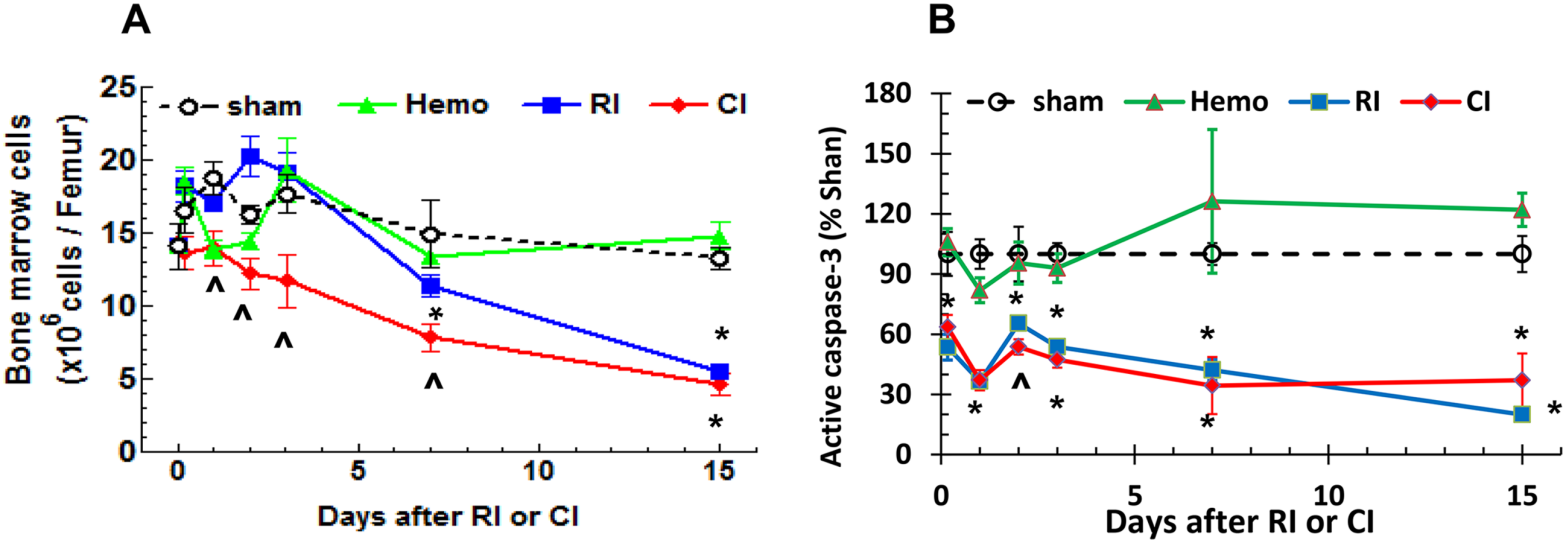
CI enhances bone marrow cell loss and whole caspase-3 increase. **(A)** Bone marrow cells from femur were counted. **(B)** Bone marrow cell lysates on day 1 were immunoblotted for detecting whole caspase-3 protein (N = 6 per group at each time point). **(C)** Bone marrow cell lysates on day 1 were assayed for measuring activated caspase-3 protein (N = 6 per group at each time point). All values are means ± SEM. *p<0.05 vs. sham and Hemo; **p<0.05 vs. respective RI time point, determined by 2-way ANOVA and Bonferroni’s inequality for panel A and Student’s *t*-test for panels B and C. Sham: free of any injury; Hemo: 20% bleeding; RI: radiation injury; CI: RI+Hemo.

Because RI [25] alone or R-W CI [26] injured bone marrow cells and reduced its cellularity, histological evaluation on sternums and cell counts of femoral bone marrows of mice were conducted. Microscopic evaluation of sternal bone marrow performed by a board certified veterinary pathologist demonstrated that both RI and CI significantly reduced bone marrow cellularity by day 3 (Fig 5A) with continued disruption through day 7 (Fig 5B). There appears to be no difference between RI and CI on microscopic examination as both are essentially acellular. However, bone marrow cells within the femur counted in an automatic cell counter (Fig 6A) demonstrated significant decreases in bone marrow cell counts after RI from days 7 through 15 (-23% to -65% vs. Sham; p<0.05). These decreases in bone marrow cell counts were further reduced by CI from days 2–7 (-48% to -24% vs. RI; p<0.05). But this CI-induced enhancement of bone marrow cell depletion disappeared by day 15.

To determine whether a reduction in bone marrow cellularity was, in part, due to enhanced caspase-dependent cell apoptosis, we measured activated caspase-3 protein levels from bone marrow cell lysates from femora (Fig 6B). Caspase-3 protein levels were significantly decreased beginning 4–5 h after RI and CI, and remained decreases up to day 15 after RI and CI. On day 2, CI further decreased activated caspase-3 protein levels. Hemo alone did not alter caspase-3 activation compared to that observed in kidney lysates of sham-operated animals (Fig 6B).

### CI enhances erythropoietin (EPO) elevation in plasma and kidney

**Fig 7:**
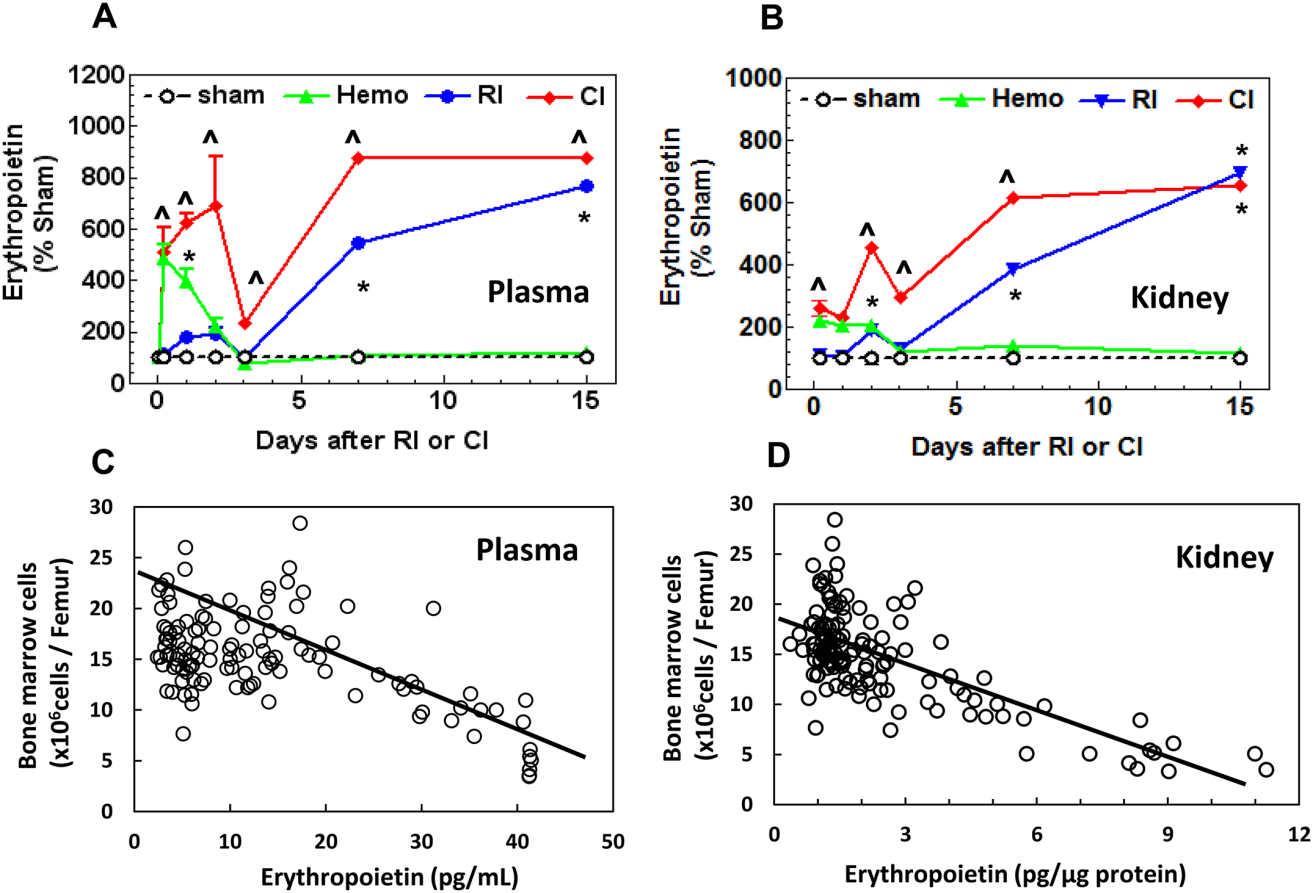
CI enhances EPO elevation in plasma and kidney. Mice received 8.75 Gy. Blood and kidney were collected at various time points (N = 6 per group at each time point). **(A)**—**(B)**, EPO in kidney lysates and plasma was measured. All values are means ± SEM. *p<0.05 vs. sham and Hemo; ^p<0.05 vs. respective RI time point, determined by 2-way ANOVA and Student’s *t*-test. **(C)** Correlation between bone marrow cell counts and EPO levels in plasma of all individual mice. R = 0.567; F = 54.93 (DFn = 1, 116; N = 118, p<0.0001), determined by linear regression. **(D)** Correlation between bone marrow cell counts and EPO levels in kidneys of all individual mice. R = 0.698; F = 127.7 (DFn = 1, 134; N = 136, p<0.0001), determined by linear regression. Sham: free of any injury; Hemo: 20% bleeding; RI: radiation injury; CI: RI+Hemo.

To determine whether CI would further increase RI-induced EPO concentrations in plasma, as previously demonstrated [27], we measured its concentrations at various time points. Hemo resulted in higher serum EPO concentrations on days 1 (+399%) and 2 (+223%) vs. Sham, but then returned to baseline levels by day 3 (Fig 7A). RI led to greater serum EPO concentrations within 4 hours post-irradiation (+117% vs. Sham, and on days 1 and 2 (+181% to +195% vs. Sham), yet also returned to the initial levels by day 3. Interestingly, serum EPO concentrations were again higher vs. Sham and Hemo groups by day 7 (+548%) and continued to be elevated through day 15 (+875%). Similarly, mice exposed to CI also demonstrated a bi-phasic elevation of EPO concentrations in serum, but these changes were significantly greater than RI alone (+130% to +496%). A similar response of EPO to RI and CI was observed in kidney lysates (Fig 7B). The increase in EPO concentrations was inversely proportional to the decrease in bone marrow cells (Fig 7C and 7D). The reduction in bone marrow cells elevated EPO concentrations in serum and kidneys to trigger the RBC production.

### Hemorrhage enhances RI-induced HIF-1α protein increases in kidney

**Fig 8:**
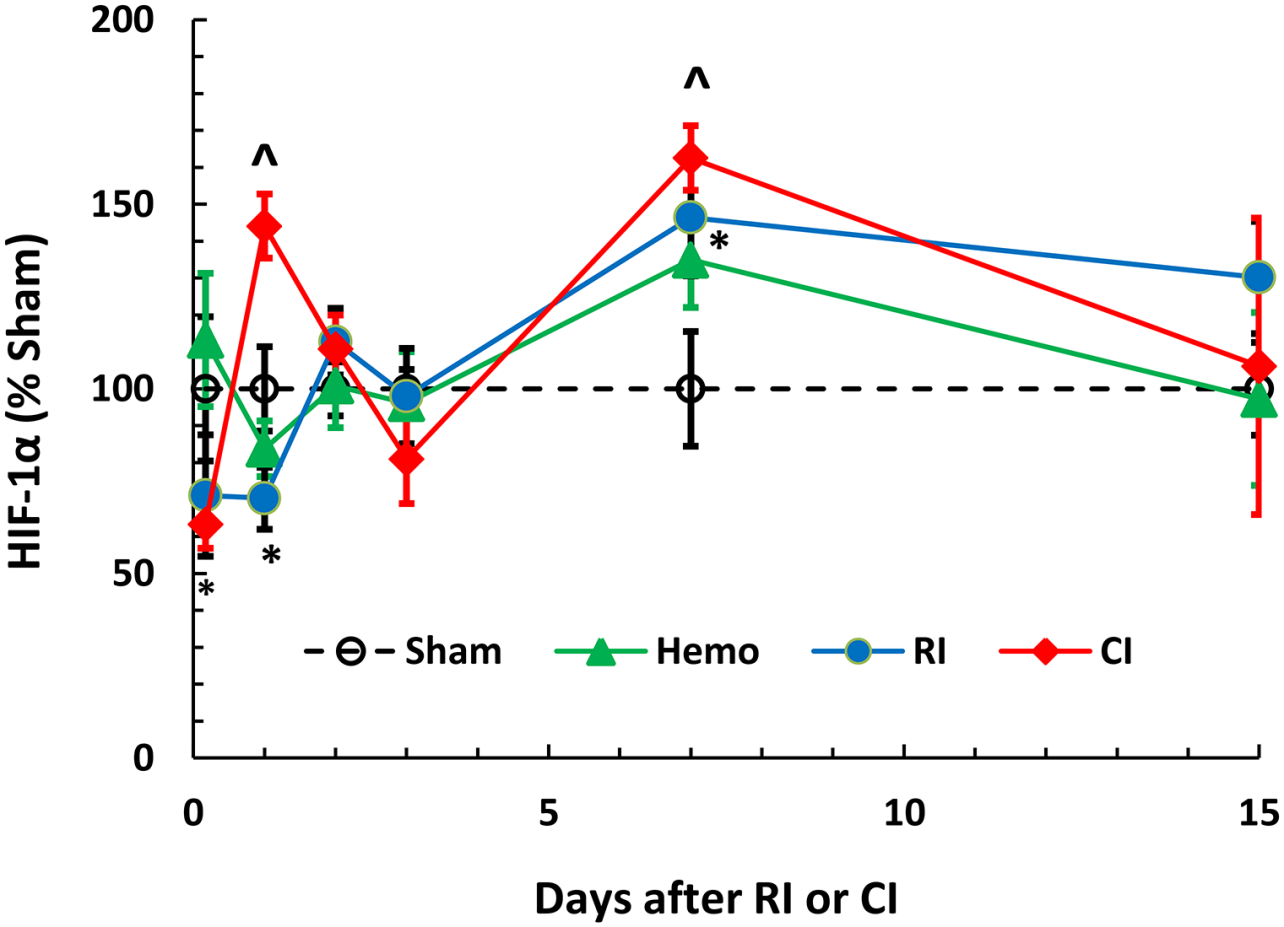
Hemorrhage enhances radiation-induced HIF-1α protein elevation in kidney. Mice received 8.75 Gy. HIF-1α protein levels in kidney at various time points after RI were measured (N = 6 per group). All values are means ± SEM. *p<0.05 vs. sham; ^p<0.05 vs. RI, determined by 2-way ANOVA and Student’s *t*-test. Sham: free of any injury; Hemo: 20% bleeding; RI: radiation injury; CI: RI+Hemo.

Hypoxia-inducible factor 1-alpha (HIF-1α) in the kidneys is activated during times of low oxygen availability, such as decreases in serum RBC levels [28]. Therefore, HIF-1α in kidneys of mice after CI was determined. Both Hemo and RI failed to increase HIF-1α protein until day 7 (+135%; 146% vs. Sham, p<0.05). In Hemo mice, the elevated HIF-1α levels returned to the basal levels by day 15, whereas after RI, the greater HIF-1α concentrations were sustained up to day 15 (+130% vs. Sham, p<0.05). Unlike Hemo or RI, CI resulted in greater kidney HIF-1α concentrations on days 1 (+144%) and 7 (+163%) as compared to RI mice, but returned to the Sham levels by day 15 (Fig 8).

### CI increases NF-κB-p50 and -p65 in kidney

**Fig 9:**
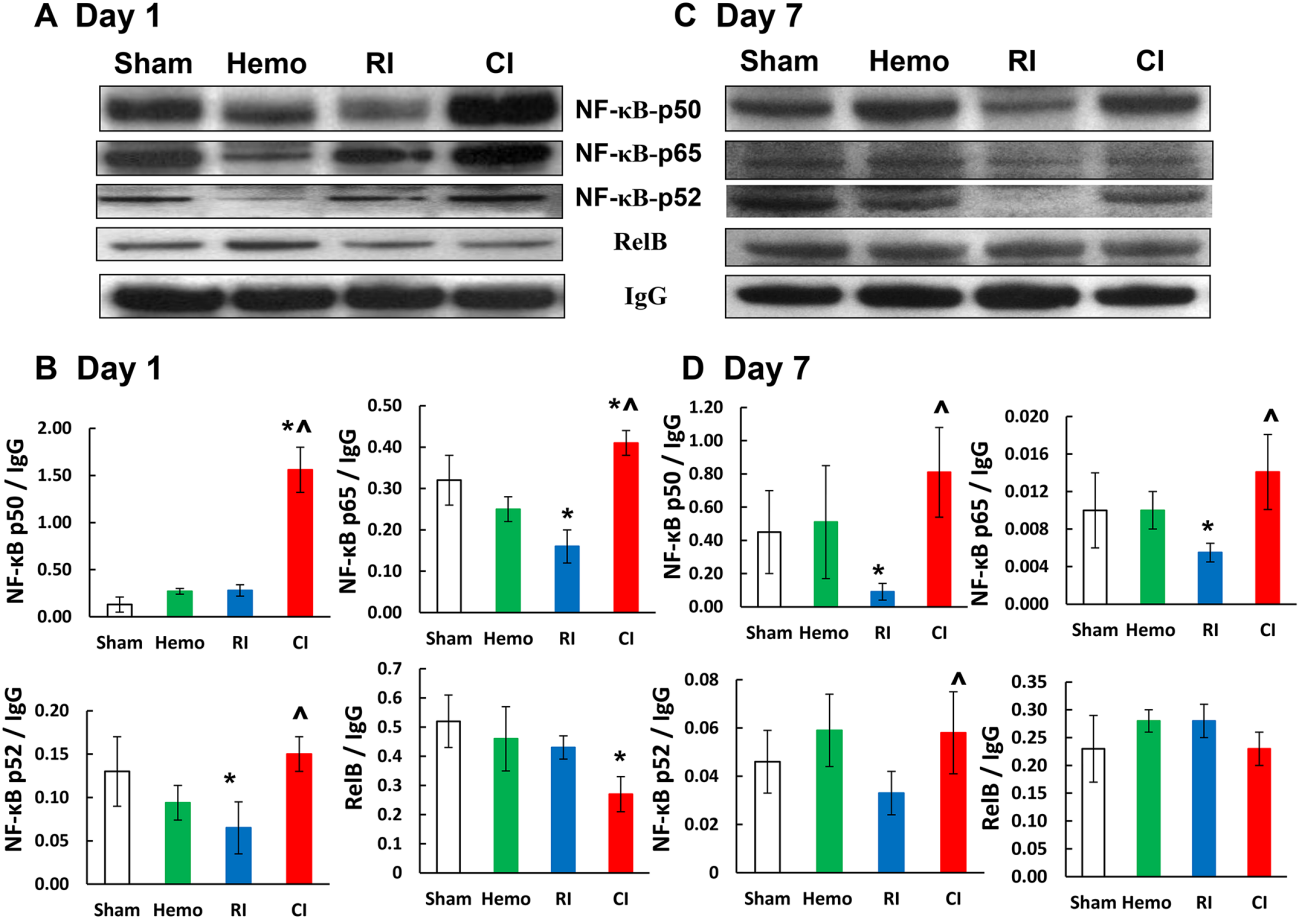
CI increases NF-κB in kidney. Mice received 8.75 Gy. Kidney lysates 1 day and 7 day after RI were immunoblotted to detect NF-κB-p50, -52, -65 and RelB (N = 6 per group). The specific bands were quantitated densitometrically. All values are means ± SEM. *p<0.05 vs. sham; ^p<0.05 vs. RI, determined by Student’s *t*-test. Sham: free of any injury; Hemo: 20% bleeding; RI: radiation injury; CI: RI+Hemo.

HIF-1α is transcriptionally regulated by nuclear factor kappa B (NF-κB) [29], we, therefore, sought to analyze NF-κB in kidney samples on day 1 (Fig 9A and 9B) and day 7 (Fig 9C and 9D) using immunoblotting. CI appeared to induce increases in p50 and p65 subunits as compared to RI mice on days 1 and 7, whereas RI alone reduced p65 on day 1 and p50 and p65 on day 7. RI alone reduced p52 only on day 1, whereas CI increased p52 on days 1 and 7. CI reduced RelB on day 1 but did not alter it on day 7.

### CI further increases RI-induced cytokine/chemokine concentrations in serum, bone marrow, ileum, spleen, and kidney

**Fig 10:**
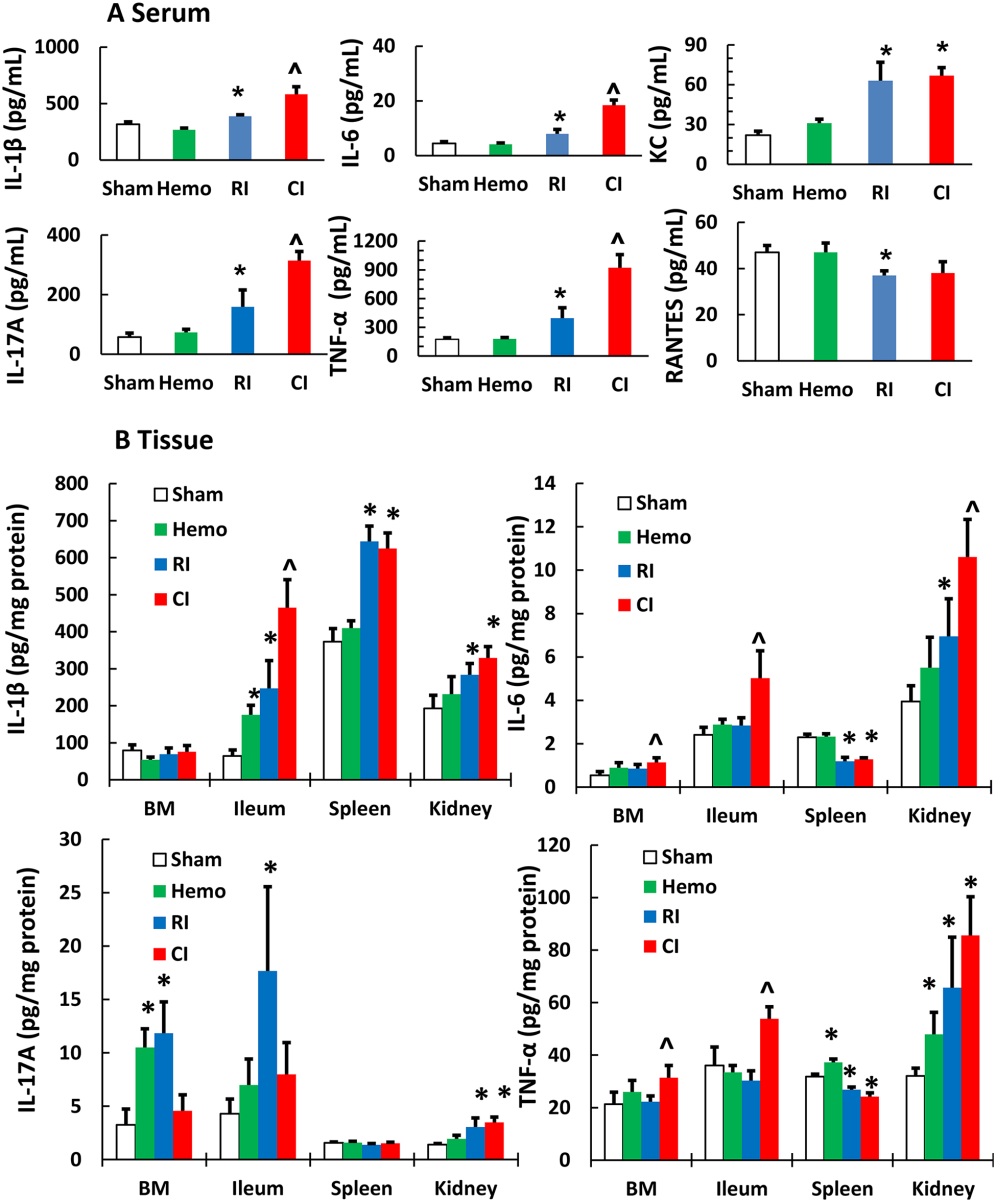
CI increases cytokines more than RI. Mice received 8.75 Gy. Serum **(A)** and tissues **(B)** including bone marrow cells, ileum, spleen, and kidney 1 day after RI and CI were assayed to measure IL-1β, IL-6, IL-17A, and TNF-α concentrations (N = 6 per group). All values are means ± SEM. *p<0.05 vs. sham; ^p<0.05 vs. RI, determined by Student’s *t*-test. Sham: free of any injury; Hemo: 20% bleeding; RI: radiation injury; CI: RI+Hemo; BM: bone marrow.

It is known that several pro-inflammatory cytokines and chemokines, such as IL-1, -6, -8, TNF-α, and RANTES produced upon NF-κB activation [30]. Therefore, the cytokines/chemokines in serum of RI and CI mice after RI or CI were measured. RI increased IL-1β, IL-6, IL-17A, TNF-α, KC (i.e., IL-8 in human), and Rantes concentrations. The increases were returned to the basal levels on day 15 (S3 Fig). CI further increased IL-1β, IL-6, IL-17A, and TNF-α on day 1 (Fig 10A).

Since these cytokines are known to be produced by macrophages, osteoblasts, smooth muscle cells, fat cells, T cells, monocytes, epithelial cells [31–35], tissue lysates of bone marrow, ileum, spleen, and kidney were evaluated for these cytokines. Fig 10B shows that Hemo significantly increased IL-1β from ileum, IL-17A from bone marrow and kidney, and TNF-α from spleen and kidney. RI increased IL-1β from ileum, spleen, and kidney, IL-6 from kidney, IL-17A from bone marrow, ileum, and kidney, and TNF-α from kidney. CI enhanced IL-1β from ileum, IL-6, from bone marrow, ileum, and kidney, and TNF-α from bone marrow and ileum.

### CI alters miRNA expressions in Kidney and their association with hematopoiesis and inflammation

**Fig 11:**
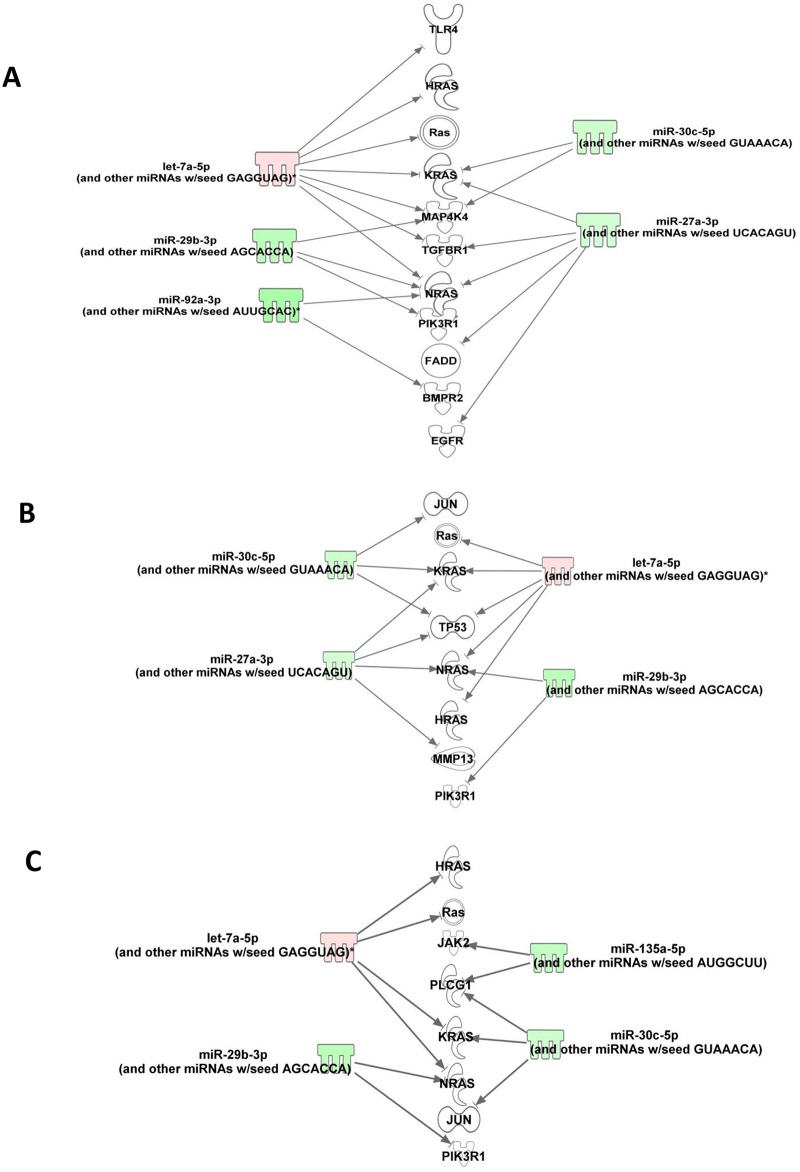
CI alters the miRNAs. Ingenuity Pathway Analysis filtered experimentally validated and direct gene targets of **(A)** NF-κB, **(B)** HIF-1, and **(C)** EPO signaling.

The miRNA expression profiling in kidney on day 1 after Hemo, RI, and CI was conducted because CI enhanced decreases in bone marrow and increases in EPO, cytokines/chemokines, and NF-κB at this time point. The miRNA expression profiling identified 29 miRNAs which were differentially expressed at 24 h after CI in kidney and among them 14 and 15 miRNAs were up- and down- regulated, respectively (Table 1). Analysis of these miRNAs for their mRNA targets with particular reference to hematopoiesis and inflammatory responses in kidney were carried out using Ingenuity Pathway analysis (IPA) program. Based on experimentally validated and computationally predicted targets information that is available at IPA, 21 out of 29 altered miRNAs targeted 9064 mRNAs. These targets were further filtered out for NF- kB, HIF-1 and EPO signaling as CI induced increases in Kidney EPO, HIF-1 and NF-κB levels mentioned above. IPA showed 6 miRNAs correlated with 18 experimentally validated and direct mRNA targets that are involved in NF-κB, HIF-1 and EPO signaling pathways. Only miRNA let-7e is upregulated among the filtered 6 miRNAs. Among the 18 experimentally validated and direct targets, five molecules such as Ras protein and their genes (Kras, Hras, and Nras) and PIK3R1 showed their association with all three NF-κB, HIF-1 and EPO signaling pathways (Fig 11, Table 2). We also found 4 miRNAs which were significantly modulated against all the three types of injuries i.e., CI, Hemo and RI (S1 Table) and showed similar expression patterns. MiRNAs viz. miR-22*, -34c* and -200b* were upregulated against all the injuries whereas expression of miR-191* was repressed by all the injury types.

**Table 1:** miRNAs in kidney of mice 24 h after CI.

Name of miRNA	Fold change	P value
**miR-425**	4494940.72	0.0000002
**miR-667**	86995.41	0.0000002
**let-7e**	10.57	0.002
**miR-331-5p**	8.97	0.004
**miR-215**	7.44	0.004
miR-322*	5.11	0.0003
**miR-487b**	3.45	0.027
**miR-494**	3.27	0.035
miR-744*	3.03	0.014
miR-181a-1*	2.83	0.007
**let-7c**	2.75	0.015
miR-214*	2.38	0.009
let-7c-1*	2.14	0.002
**miR331-3p**	2.07	0.01
**miR-218**	-1.96	0.007
**miR-101b**	-1.96	0.031
**miR-27a**	-1.96	0.005
**miR-194**	-2.04	0.011
**miR-450a**	-2.09	0.041
**miR-30e**	-2.19	0.004
**miR-598**	-2.43	0.011
**miR-190**	-2.48	0.004
**miR-379**	-2.65	0.027
**miR-135a**	-2.66	0.02
**miR-363**	-2.81	0.003
**miR-29b**	-2.94	0.001
**miR-337-5p**	-3.2	0.019
miR-322*	-3.94	0.006
**miR-193**	-35.52	0.03

**Table 2:** IPA NF-kB, HIF-1 and EPO filtered miRNAs and their experimentally validated and direct gene targets. Let-7e targets *RAS*, *HRAS*, *KRAS*, and *NRAS* that are common for NF-κB, HIF-1 and EPO singling. miR-29b targets *NRAS* and *PIK3R1* that are also common for NF-κB, HIF-1 and EPO singling. miR-30e targets *KRAS* for NF-κB, HIF-1 and EPO singling and *JUN* for only HIF-1 and EPO singling. miR-27a targets *KRAS* and *NRAS* for only NF-κB and HIF-1 signaling. miR-32 targets NRAS for only NF-κB signaling.

S#	miRNA	NF-κB	HIF-1	EPO
**1**	**Let-7e**	*RAS*	*RAS*	*RAS*
**2**		*HRAS*	*HRAS*	*HRAS*
**3**		*KRAS*	*KRAS*	*KRAS*
**4**		*NRAS*	*NRAS*	*NRAS*
**5**		*MAP4K4*		
**6**		*TGFBR1*		
**7**		*TLR4*		
**8**			*TP53*	
	**miR-29b**	*NRAS*	*NRAS*	*NRAS*
**9**		*PIK3R1*	*PIK3R1*	*PIK3R1*
		*MAP4K4*		
	**miR-30e**	*KRAS*	*KRAS*	*KRAS*
**10**			*JUN*	*JUN*
		*MAP4K4*		
**11**				*PLCG1*
			*TP53*	
	**miR-27a**	*KRAS*	*KRAS*	
		*NRAS*	*NRAS*	
**12**		*EGFR*		
**13**		*FADD*		
**14**		*TGFBR1*		
			*TP53*	
**15**			*MMP13*	
	**miR-32**	*NRAS*		
**16**		*BMPR2*		
**17**	**miR-135**			*JAK2*
**18**				*PLCG1*

## Discussion

The current study presents evidence that CI (RI+Hemo) increases mortality compared to RI (Fig 1A) and triggers a reduction in body weight and water intake (Figs 1D and 2A). The DMF comparing the LD_50/30_ for CI and RI was 1.046. Animal studies with several animal species have demonstrated similar results with other types of CI [13, 36–42]. In dogs, exposure to 100 roentgen radiation caused 0% mortality and 20% total body surface area (TBSA) burns alone were non-lethal; whereas the combination of burns and radiation elicited 73% mortality [39]. Similarly, in rats given 33% TBSA burns, baseline mortality was 7% [36]. However, when the burns were combined with 250 roentgen radiation, mortality was increased to 86% [36]. In mice given 15% TBSA wounds or burns, baseline mortality was 2.8–5.6%. But when 3 Gy (94% neutron and 6% γ photons) or 7 Gy (100% γ photons) were combined with wounds or burns, the combined injury resulted in 22–83% and 15–60%, respectively, depending on the time intervals between the radiation and wounds or burns [5, 6]. The current experimental model of 20% Hemo in combination with sub-lethal RI also resulted in a similar synergistic mortality. Our results suggest that mortality may be associated with body weight reduction combined with other factors including GI injury and systemic bacterial infection (Kiang, unpublished data) but not water intake. The significant body weight reduction in CI mice occurred by day 15 (Fig 2A). Water intake by CI mice was the least of all experimental groups on days 2, 5, and 6, followed by RI and then Hemo and Sham, respectively, but returned almost to normal volumes on day 7 (Fig 1D). Since RI and CI mice still died later even though they drank similar amount of water as sham mice, it is thought that water intake is not associated with the mortality. However, Hemo did not alter GI weight, but transiently elevate spleen weights. By day 15, RI and CI mice exhibited similar reductions in organ weights, suggesting that the greater body weight reduction after CI could be contributed by other organs.

Hemo alone significantly increased neutrophils and platelets but did not alter other blood cell counts; the phenomenon was similar to that after wounding alone because of the need of wound healing [8]. Like wounds or burns in combination with RI, CI (i.e., RI+Hemo) triggered a greater blood cell loss than RI alone (Figs 3 and 4), a synergistic effect. The decreased activated caspase-3 protein in bone marrow after RI and CI suggests caspase-dependent cell apoptosis may not happen. CI probably induces more cell death through involvement in necrosis or autophagy. This view was confirmed with 1) the observation of decreased femur bone marrow cell counts and 2) that RI and CI did not increase activated caspape-3 protein level in bone marrow but had low bone marrow cellularity on microscopic examination.

Reductions in RBC or hemoglobin trigger EPO production that is mediated by HIF-1α binding to a 50-bp hypoxia-inducible enhancer that is approximately 120 bp 3' to the polyadenylation site [28, 43]. EPO acts 1) primarily to rescue erythroid cells from apoptosis to increase their survival, and 2) synergistically with growth factors including SCF, GM-CSF, 1L-3, and IGF-1 to induce maturation and proliferation of erythroid progenitor cells [43]. Herein, we observed that RI induced biphasic increases in HIF-1α in kidney and EPO in plasma and kidney. The data are in agreement with published data [27]. CI transiently enhanced these increases, a synergistic effect, which was corresponded to the bone marrow cell loss. The mechanism underlying the CI-induced enhancement is not clear. Because the CI-induced enhancement in HIF-1α increases occurred on days 1 and 7, the enhancement observed on day 1 could be attributed by radiation-induced free radical production [44], while the enhancement found on day 7 could be due to the RBC reduction (Fig 4A–4C).

HIF-1α is transcriptionally regulated by NF-κB-p50 and—p65 in kidneys [29]. RI alone significantly decreased NF-κB-p50 on day 7 and—p65 in kidneys on days 1 and 7 after RI. In contrast, CI significantly increased both proteins on days 1 and 7, although the cause of these elevations is not yet apparent but could be simply due to elevation of NF-κB-p50 and—p65 (Fig 9), resulting in the transiently increased HIF-1α expression at these time points (Fig 4).

It is known that several pro-inflammatory cytokines and chemokines, such as IL-1, -6, -8, TNF-α, and RANTES produced upon NF-κB activation [30]. Our preliminary data have been demonstrated that they were synergistically increased after CI in serum, bone marrow, ileum, spleen, and kidney, in agreement with published observations [45], further confirming the relationship between NF-κB and cytokines/chemokines. IL-17 is known to induce production of IL-1 and TNF-α from many cell types [46, 47].

MiRNAs are short RNA molecules of 20–25 nucleotides in length that negatively regulate gene expression in animals and plants primarily by targeting 3’ untranslated regions of mRNAs.

One of the goals in the present study was to understand the early changes occurring in kidney at the molecular level after CI in order to identify the molecular targets which may be helpful to initiate early treatment of CI. We identified several miRNAs which were either uniquely modulated against CI only or commonly modulated against all the three types of injuries in the kidney. MiR-22*, -34c*, -191* and -200b* were found to be significantly modulated against CI, Hemo and RI; however, nothing much is known about their role in Hemo, RI or CI at present and thus warrants further studies in future. Although there have been several studies evaluating effects of Hemo or RI on miRNA expression, we were also interested to identify any unique effects of CI alone on miRNA expression. CI altered three miRNAs involved in NF-κB, EPO and HIF-1 signaling. Significantly altered expression of let-7 family has been shown in response to radiation by several miRNA profiling studies [48]. However, radiation exposure to various cell lines showed inconsistencies such as upregulation and downregulation of let-7 miRNAs expressions [48]. Increased let-7 in mouse hematopoietic stem cells showed marked reduction in their self-renewal activity [49]. Pathway analyses indicate Ras protein and three ras genes (Kras, Hras and Nras) is the target of upregulated let-7e. Lower level of let-7 expression increased the expression of Ras protein in pathogenesis of human lung cancer [50]. The Ras family of genes is involved in the cellular regulation of proliferation, differentiation, cell adhesion and apoptosis. One of the Ras genes, Kras was reported as a well-established target of let-7 and also demonstrated to be a part of the MAPK signaling pathway [51] Upregulated let-7 in response to CI may damage the kidney blood cells that involved in proliferation and differentiation when exposed to radiation [48]. Acute kidney injury induces NF-κB, one of the major inflammation mediators and also the transcriptional factor HIF-1 that involved in the cellular response to low level of oxygen, hypoxia [52, 53]. Recently, the HIF-1 has been shown to play a role in inflammation, indicating that both HIF-1 and NF-κB pathways were intimately linked. These two major pathways share common target genes including EPO that is produced by the kidney which regulates red blood cell production in the bone marrow as indicated in our bioinformatics analysis. Studies have also reported physical interaction between these molecules [53, 54]. NF-kB has been shown to directly activate let-7e during osteoclast differentiation [55]. The association between let-7e and Ras protein and all three Ras genes in CI indicates that it may play a significant role in NF-κB-HIF-1-EPO cascade.

Two downregulated miRNAs, viz. miR-29b, and miR-30e in CI may be involved in induced inflammatory damage by activating NF-κB signaling pathway and regulation of osteoblast differentiation [56–58]. MiR-29b was reduced by 2.94 fold (Table 1) and is known to be directly repressed by NF-κB, while indirectly induced by NF-κB through HDAC [30]. Likewise, CI decreased miR-30e by 2.19 fold (Table 1). Zu et al. [59] reported that miR-30e decrease led to hyperactive NF-κB. Indeed, our data is in agreement with their observations. Using IPA program, Fig 10 shows that miR-30e reduction leads to multifactorial changes including IL-1 and TNF-α. The relationship of miR-29b and -30e to HIF-1α is unclear and warrants an exploration. CI increased miR-425 and miR-667 many-fold that should be explored as well. Nevertheless, suppression of miR-30 and IL-1β protects CD2F1 male mice and human CD34+ cells from radiation injury [45]. Knockdown of NF-κB-p65 by small interfering RNA (siRNA) significantly suppresses radiation-induced miR-30 expression in CD34+ cells [45].

Either RI or Hemo have been reported to increase inducible nitric oxide synthase (iNOS) [4, 16] and p53 activation, molecules known to contribute to cell apoptosis [4, 60, 61]. Possibilities of activation of iNOS and p53 involved in the mortality after CI cannot be excluded. Additionally, it is evident that AKT and MAPK signaling network has also been altered by RI [62] and Hemo [63]. Therefore, alteration of the AKT and MAPK signaling network by CI is highly likely and require further investigation, which is under way in this laboratory.

In summary, we report that Hemo reduced the LD_50/30_ from 9.1 Gy (RI) to 8.7 Gy (CI) with a DMF = 1.046. RI resulted in deleterious effects on leukocytopenia, thrombopenia, erythropenia, and bone marrow cell depletion, probably not via activation of caspase-dependent apoptosis. RI increased some of cytokines/chemokines. Hemo transiently exacerbated these deleterious effects. EPO production via HIF-1α was increased in kidneys and blood after both RI and CI, although CI mice exhibited more significant effects. CI also significantly reduced miR-29b and -30e along with other miRNAs which have been shown to result in increased inflammatory responses. This study provides preliminary evidence that Hemo exacerbates RI-induced mortality and cell losses associated with high-dose γ-radiation. We were also able to identify some of the early changes occurring due to CI which may assist in starting an early treatment regimen before the onset of late effects of CI. However, further studies are warranted to confirm the role played by these miRNAs and proteins in enhancement of CI-induced mortality. Taken together, we have identified some of the physiological and molecular changes occurring during CI which may have facilitated in worsening the injury and hampering the recovery of animals ultimately resulting in higher mortality.

## Supporting Information

S1 ARRIVE Checklist The ARRIVE Guidelines Checklist. Animal Research: Reporting in vivo experiments.
(PDF)

S1 Fig Water consumption after hemorrhage. Mice received 20% hemorrhage (Hemo). The average daily consumption sham animals in this study was 3.929±0.095 mL/mouse/day. N = 7 per group per time point. *p<0.05 vs. sham at the time point, determined by Student’t- t-test.
(TIF)

S2 Fig Body weights monitored at various time points after sham control, hemorrhage, irradiation, and irradiation followed by hemorrhage. Mice were irradiated at 8.75 Gy followed by 20% hemorrhage. Then their body weights were measured at various time points after hemorrhage (Hemo), irradiation (RI), or RI followed by Hemo (CI). Data were pooled from three separate experiments with N = 3–35 per time point per group. *p<0.05 vs. sham; ^p = 0.08 vs. RI, determined by Student’s t-test.
(TIF)

S3 Fig Changes in cytokine/chemokine concentrations after sham control, hemorrhage, irradiation, and irradiation followed by hemorrhage. Mice were irradiated at 8.75 Gy followed by 20% hemorrhage. Then serum were collected at various time points after hemorrhage (Hemo), irradiation (RI), or RI followed by Hemo (CI). Cytokine/chemokine concentrations in serum were measured. N = 6 per group per time point per group except N = 3 of CI group on day 15. ^p<0.05 vs. RI, determined by Student’s t-test.
(TIF)

S1 Table miRNAs modulated by hemorrhage, irradiation, and irradiation followed by hemorrhage in kidney. Mice were irradiated at 8.75 Gy followed by 20% hemorrhage. miRNAs in kidneys on day 1 after hemorrhage (Hemo), irradiation (RI), or RI followed by Hemo (CI) were profiled (N = 4 per group).
(DOC)
