# Supplementary figures and images for: Hemorrhage Exacerbates Radiation Effects on Survival, Leukocytopenia, Thrombopenia, Erythropenia, Bone Marrow Cell Depletion and Hematopoiesis, and Inflammation-Associated microRNAs Expression in Kidney
Source: PLoS One. 2015 Sep 30;10(9):e0139271. doi: 10.1371/journal.pone.0139271 (PMC4589285; doi:10.1371/journal.pone.0139271)

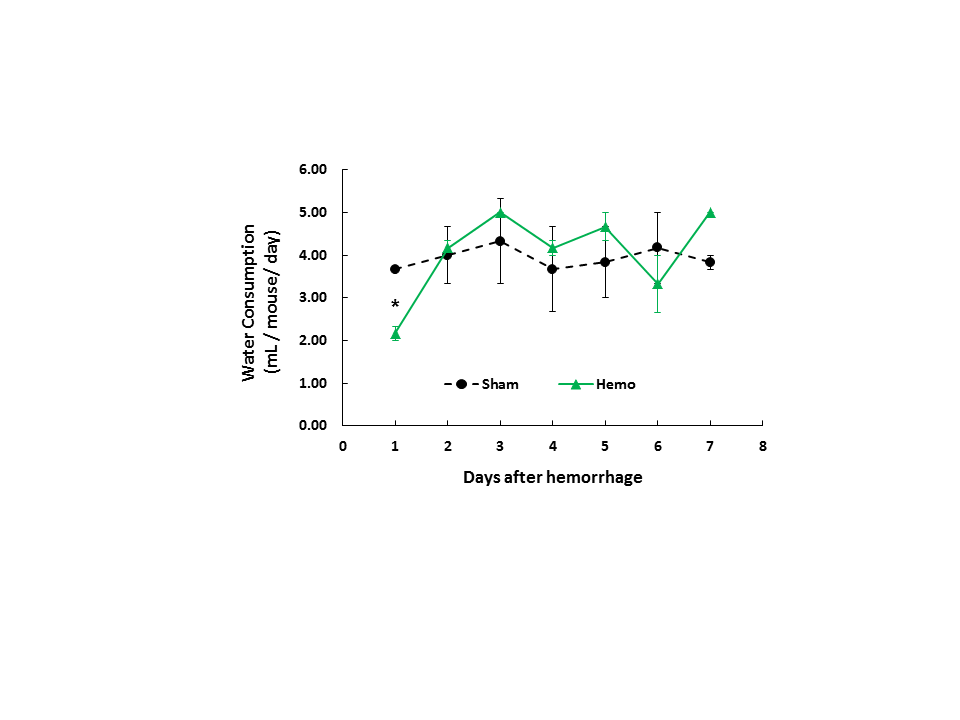

Supplement: S1 Fig — Mice received 20% hemorrhage (Hemo). The average daily consumption sham animals in this study was 3.929±0.095 mL/mouse/day. N = 7 per group per time point. *p<0.05 vs. sham at the time point, determined by Student’t- t-test. (TIF) [file pone.0139271.s002.tif]

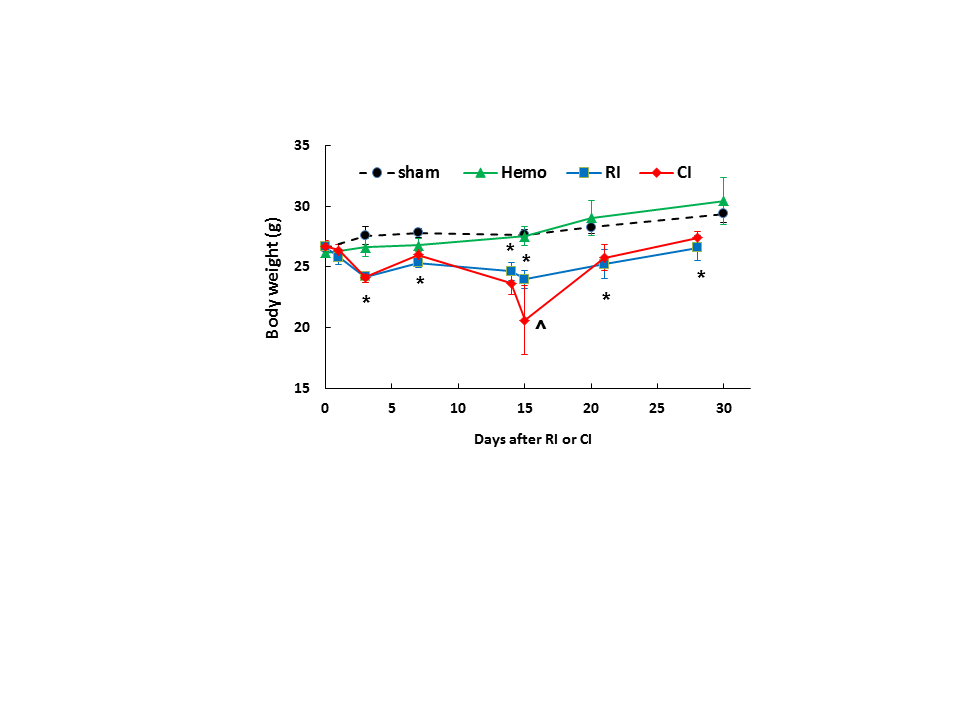

Supplement: S2 Fig — Mice were irradiated at 8.75 Gy followed by 20% hemorrhage. Then their body weights were measured at various time points after hemorrhage (Hemo), irradiation (RI), or RI followed by Hemo (CI). Data were pooled from three separate experiments with N = 3–35 per time point per group. *p<0.05 vs. sham; ^p = 0.08 vs. RI, determined by Student’s t-test. (TIF) [file pone.0139271.s003.tif]

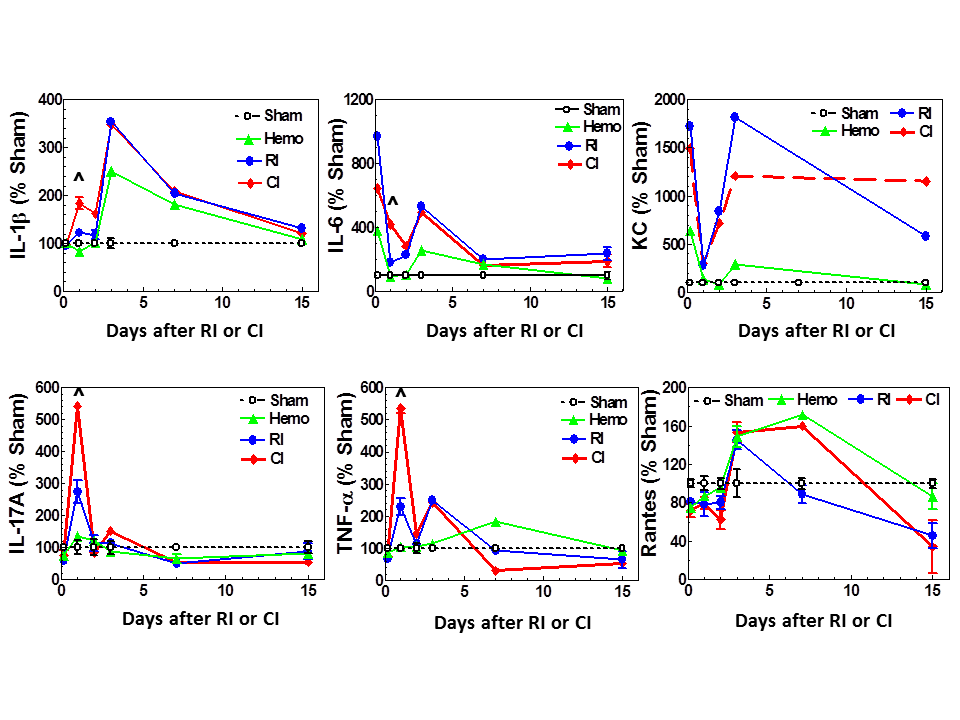

Supplement: S3 Fig — Mice were irradiated at 8.75 Gy followed by 20% hemorrhage. Then serum were collected at various time points after hemorrhage (Hemo), irradiation (RI), or RI followed by Hemo (CI). Cytokine/chemokine concentrations in serum were measured. N = 6 per group per time point per group except N = 3 of CI group on day 15. ^p<0.05 vs. RI, determined by Student’s t-test. (TIF) [file pone.0139271.s004.tif]
