# Supplementary material for: Hemorrhage Exacerbates Radiation Effects on Survival, Leukocytopenia, Thrombopenia, Erythropenia, Bone Marrow Cell Depletion and Hematopoiesis, and Inflammation-Associated microRNAs Expression in Kidney
Source: PLoS One. 2015 Sep 30;10(9):e0139271. doi: 10.1371/journal.pone.0139271 (PMC4589285; doi:10.1371/journal.pone.0139271)
Supplement: S1 Table — Mice were irradiated at 8.75 Gy followed by 20% hemorrhage. miRNAs in kidneys on day 1 after hemorrhage (Hemo), irradiation (RI), or RI followed by Hemo (CI) were profiled (N = 4 per group). (DOC) [file pone.0139271.s005.doc]

**S1 Table: miRNAs modulated by all the three types of injuries in kidney of mice at** 24 h.

| **Name of miRNA** | **CI** | | **Hemo** | | **RI** | |
| --- | --- | --- | --- | --- | --- | --- |
|  | **Fold Expression** | **P-value** | **Fold Expression** | **P-value** | **Fold Expression** | **P-value** |
| **miR-200b*** | 8.79 | 0.023 | 7.17 | 0.034 | 17.07 | 0.008 |
| **miR-22*** | 3.55 | 0.0003 | 3.69 | 0.0002 | 9.21 | 0.0001 |
| **miR-34c*** | 2.25 | 0.019 | 2.71 | 0.006 | 2.10 | 0.003 |
| **miR-191*** | -3.21 | 0.001 | -4.11 | 0.001 | -2.20 | 0.008 |
